# Relationship between Plasma Aldosterone Levels and Left Ventricular Mass in Hypertensive Africans

**DOI:** 10.1155/2013/762597

**Published:** 2013-10-07

**Authors:** Adewole Adebiyi, Olubayo Akinosun, Chibuike Nwafor, Ayodele Falase

**Affiliations:** ^1^Department of Medicine, College of Medicine, University of Ibadan, Ibadan 200212, Nigeria; ^2^Department of Chemical Pathology, College of Medicine, University of Ibadan, Ibadan 200212, Nigeria

## Abstract

*Background.* Hypertension is the most common cardiovascular disease worldwide and is a major cause of morbidity and mortality. Studies have suggested that the activity of the renin-angiotensin-aldosterone system play a major role in the target organ damage such as left ventricular hypertrophy occuring in hypertension. We sought to determine the relationship between plasma aldosterone and left ventricular mass in untreated African hypertensives. *Methods. *We recruited 82 newly diagnosed and untreated hypertensives and 51 normal controls. Measurements obtained included echocardiographic LV mass index, plasma aldosterone and renin. *Results.* The hypertensive subjects had lower renin levels (21.03[6.974] versus 26.66[7.592] ng.mL^−1^, *P* = 0.0013), higher LV mass index (52.56[14.483] versus 42.02[8.315] g.m^−2.7^ *P* < 0.0001) when compared with the controls. There were no univariate associations between LV mass index and plasma aldosterone (*r* = 0.0179, *P* = 0.57) and between LV mass index and plasma renin (*r* = 0.0887, *P* = 0.61). In a multivariate model involving LV mass index and age, sex, body mass index (BMI), plasma aldosterone, plasma renin and systolic blood pressure (SBP), only age (*P* = 0.008), BMI (*P* = 0.046), and SBP (*P* = 0.001) were independently associated with the LV mass index. *Conclusions*. In this group of hypertensive Africans, there is no independent association of plasma aldosterone with LV mass. The height of the blood pressure, the body mass index and the age of the subjects determined the LV mass.

## 1. Introduction

 Hypertension is a major cause of morbidity and mortality worldwide [1]. Hypertension is more severe and associated with more severe sequelae in blacks when compared with subjects of Caucasian extraction [2–5]. Studies have also shown that black subjects have lower plasma renin levels [6–8]. It is thought that black subjects have inappropriately elevated levels of aldosterone relative to plasma renin activity [9]. The activity of the renin-angiotensin-aldosterone system (RAAS) is thought to be the major contributor to the pathogenesis of hypertension and its sequelae [10]. Studies have shown that circulating aldosterone levels within the physiologic range are strongly related to increased risk of cardiovascular mortality, fatal stroke, and sudden cardiac death [11]. It has also been shown that chronic administration of aldosterone in animal models leads to cardiac fibrosis and left ventricular hypertrophy (LVH). Subjects with primary aldosteronism have early and more severe development of LVH when compared with subjects with essential hypertension. These would suggest a blood pressure independent effect of aldosterone on the heart. Though previous studies, on predominantly white subjects, had not been conclusive on the relationship between left ventricular mass and aldosterone, very few of these studies have been done on black subjects. This study therefore attempts to determine the relationship between aldosterone levels and left ventricular mass in black subjects with essential hypertension.

## 2. Methods

### 2.1. Subjects

 Subjects were newly diagnosed but untreated hypertensive subjects recruited from the clinics of the University College Hospital, Ibadan; through community screening exercises; and from among the staff of the University College Hospital, Ibadan. Subjects with secondary hypertension, diabetes mellitus, symptomatic heart failure, and chronic kidney disease were excluded. Informed consent was obtained from each subject, and ethical approval was obtained from the joint Institutional Review Board (IRB) of the College of Medicine, University of Ibadan/University College Hospital, Ibadan, Nigeria.

### 2.2. Data Collection

 Blood samples were obtained in the morning after an overnight fast. No caffeine-containing beverages, alcohol, or smoking was allowed for at least 2 h. Subjects were not on any medications prior to sample collection. Blood was collected into test tubes on ice after 30 minutes of rest in the supine position. Plasma and serum was obtained following rapid centrifugation. A 24 h collection of urine was made the preceding day in plastic containers containing HCl (80 mmol/L final concentration). Subjects notified the start and end of the collection time. The amount of urine was quantified at return of the containers. All samples were stored at −70°C until further analyses.

### 2.3. Biochemical and Hormonal Analyses

 High-performance (cation exchange) liquid chromatography (HPLC) was used to determine the plasma renin, plasma aldosterone. The sensitivity and specificity of the HPLC assay are 98% and 92%, respectively. Plasma and urinary sodium and potassium concentrations were measured using a flame photometric method.

### 2.4. Echocardiography

 Echocardiographic examination was done using a Phillip Aloka SSD 4000 (Aloka Co. Ltd., Tokyo, Japan) machine with a 2.5–5.0 MHz linear array transducer. Measurements were made as recommended by the American Society of Echocardiography [12]. Two experienced physicians performed the echocardiography. In our laboratory, the intraobserver concordance correlation coefficient ranged from 0.76 to 0.98 while that of the interobserver concordance ranged from 0.82 to 0.96. Measurements of LV internal diameter (LVIDD), intraventricular septal thickness (IVSTD), and posterior wall thickness (PWTD) were obtained at end diastole. LV mass (LVM) was calculated using the formula published by Devereux et al. [13]: 0.8 × (1.04 × ((LVIDD + PWTD + IVSTD)3 − LVIDD3)) + 0.6, and LVM was indexed to the allometric power of height. Relative wall thickness (RWT) was measured at end diastole as the ratio 2(PWT)/LVID.

#### 2.4.1. Statistical Methods

 Data from continuous variables are presented as mean (standard deviation) or as medians with interquartile ranges while categoric data are expressed as percentages. All statistical tests were two-sided and carried out to a significance level (*P*) of 0.05. Normality of continuous variables was assessed by the Shapiro-Wilks test. Student's *t*-test for independent groups was used to compare normal continuous data while Mann-Whitney's test was used to compare nonnormal data.

Tertiles of plasma aldosterone were generated and the aldosterone groups were compared with analysis of variance for continuous data, and *χ* ^2^ test was used for categoric data. Pearson correlation analysis was used to determine the correlation between plasma aldosterone and ARR and the echocardiographic variables of LV structure. A stepwise linear regression model (with backward elimination) was used to evaluate the relationship between LV mass index and plasma aldosterone. The statistical program used was *R*, version 2.15.0 [14].

## 3. Results

 Eighty-two hypertensive and fifty-one controls were studied. The clinical characteristics of the subjects are shown in Table 1. The age and the anthropometric measures of the subjects were similar.  Table 2 shows the biochemical, hormonal, and echocardiographic parameters of the subjects. Plasma sodium and renin were lower in the hypertensive subjects. The left atrial, LV wall dimensions, and LV mass were higher in the hypertensives. The hypertensive subjects frequently had more hypertrophic LV geometric patterns when compared with the normal subjects. There were no differences in the plasma aldosterone levels and the urinary excretion of sodium and potassium.

Table 3 shows the biochemical, hormonal, and echocardiographic parameters of the subjects according to plasma aldosterone tertiles. There was no relationship between the aldosterone groups and various parameters of LV structure.

There was no univariate association between the parameters of LV structure and the plasma aldosterone and aldosterone-to-renin ratio levels (Table 4). In a multivariate model involving LV mass index and age, sex, body mass index (BMI), aldosterone, plasma renin, pulse pressure, and systolic blood pressure (SBP), only age (*P* = 0.008), BMI (*P* = 0.046), and systolic blood pressure (*P* = 0.001) were independently associated with the LV mass index (*R* ^2^ = 0.15, *P* < 0.0001).

## 4. Discussion

 In this group of black African subjects, there was no association of plasma aldosterone with LV mass. Also, no gender-specific differences in the relationship of LV mass index with plasma aldosterone were seen. Only the age of the subjects, their body mass index, and the level of their blood pressure were associated with LV mass.

Previous studies on the association between LV mass and circulating aldosterone levels in subjects with essential hypertension had yielded conflicting results. El-Gharbawy et al. [9] found no association between aldosterone levels and LV structure in hypertensive caucasians and nonobese black Americans, and Vasan et al. [15] found no relationship between LV mass and serum aldosterone in the Framingham cohort. Other studies [16–24] had noted associations between LV mass and blood aldosterone levels. These conflicting results could be due to differences in the study populations, that is, presence of other comorbidities such as diabetes mellitus, long-standing hypertension, differences in dietary salt intake, and the role of drugs that could alter the renin-angiotensin-aldosterone system. Our study subjects were newly diagnosed and treatment-naive hypertensives; subjects with elevated blood sugar and diabetes mellitus were excluded and were African subjects living in tropical environment. It is quite probable that significant racial differences exist in the relationship between aldosterone levels and LV mass.

Vasan et al. [15] had documented a significant association between plasma aldosterone levels and LV posterior wall thickness and relative wall thickness but no relationship between LV mass index and aldosterone in women, while Edelmann et al. [17] reported a robust and consistent association between echocardiographic parameters of LV structure and serum aldosterone concentration exclusively in women. These gender differences had been thought to be due to differences in adaptation to pressure overload due to oestrogen signalling in the myocardium [25]. In contrast to these studies, we did not find any gender related differences in the association of LV mass and plasma aldosterone levels. This is likely due to the differences in the study populations. It is also probable that envirnomental and dietary factors play a significant role in the modulation of the effects of the renin-angiotensin-aldosterone system in native black Africans.

Our study demonstrated an association between LV mass index and body mass index. The influence of body mass index on LV mass had been noted in previous studies [26, 27]. Mulè et al. [23] in their study among subjects with metabolic syndrome suggested that obesity might play a role in the association of LV mass with aldosterone levels. However, our study showed no relationship between body mass index and aldosterone levels.

The influence of the level of the blood pressure especially systolic blood pressure [28–30] on LV mass index had been previously described in several studies. This is supported by the findings from this study that shows the independent association of systolic blood pressure with LV mass index. The influence of systolic blood pressure on the development of left ventricular hypertrophy is thought to be a result of an increased end-systolic stress which is mostly related to the systolic blood pressure [31].

This study also demonstrates the independent association of age with LV mass index. This supports observations from previous studies that target organ damage in hypertension increases progressively with age [32, 33]. Though our patients were newly diagnosed with hypertension, many of the subjects could have had longstanding but undiagnosed hypertension. Ayodele et al. [34] working in South-West Nigeria found that about 42% of their subjects already had left ventricular hypertrophy at diagnosis of their hypertension.

Our study is limited in that only a single measurement of aldosterone was made and might be insufficient in estimating the daily exposure to aldosterone. The determination of 24 hr urinary aldosterone is better suited for the assessment of daily aldosterone load [35].

In conclusion, in our group of black newly diagnosed hypertensive subjects, we did not find an independent association of LV mass index with plasma aldosterone concentration. Age and systolic blood pressure were independently associated with LV mass in our subjects. Further studies relating the 24 hr aldosterone load might be necessary to fully evaluate the association of aldosterone and measures of LV structure.

## Figures and Tables

**Table 1:** Clinical characteristics of the subjects.

Variable	Hypertensives	Normal controls	*P* value
	(*n* = 82)	(*n* = 51)	
Age (years)	49.8 (10.96)	47.8 (11.21)	0.3396
Weight (kg)	74.6 (13.65)	73.3 (16.11)	0.4098
Height (cm)	167.3 (8.43)	165.6 (8.84)	0.2790
Body mass index (kg·m^−2^)	26.66 (4.654)	26.65 (5.134)	0.9668
Obese (%)	21.2	18.3	0.8116
Pulse (bpm)	75.3 (12.62)	71.47 (12.782)	0.0567
Systolic blood pressure (mmHg)	165.45 (15.452)	127.19 (10.401)	0.0000
Diastolic blood pressure (mmHg)	95.62 (7.137)	76.5 (7.733)	0.0000
Pulse pressure (mmHg)	69.83 (15.452)	50.69 (8.098)	0.0000
Mean arterial blood pressure (mmHg)	118.89 (7.778)	93.39 (7.833)	0.0000

**Table 2:** Biochemical, hormonal, and echocardiographic characteristics of the subjects.

Variable	Hypertensives	Normal controls	*P* value
	(*n* = 82)	(*n* = 51)	
Plasma sodium (mmol·L^−1^)	136.44 (7.874)	139.58 (6.591)	0.0328
Plasma potassium (mmol·L^−1^)	4.02 (0.801)	4.11 (0.775)	0.5814
Urinary sodium (mmol·L^−1^)	68.35 (41.173)	63.33 (40.538)	0.4968
Urinary potassium (mmol·L^−1^)	16.06 (10.544)	13.99 (10.782)	0.2036
Urinary sodium excretion (mmol·24 hr^−1^)	103.93 (63.571)	90.77 (50.873)	0.4522
Urinary potassium excretion (mmol·24 hr^−1^)	24.04 (16.527)	20.44 (14.761)	0.1863
Plasma renin (ng·mL^−1^)	21.03 (6.974)	24.66 (7.592)	0.0013
Plasma aldosterone (nmol·L^−1^)	245.82 (59.498)	262.46 (58.136)	0.1162
ARR	12.66 (5.112)	11.03 (2.657)	0.1198
Left atrial diameter (cm)	3.94 (0.511)	3.69 (0.491)	0.0070
Aortic valve opening (cm)	1.89 (0.257)	1.86 (0.243)	0.4785
LV posterior wall thickness (cm)	1.19 (0.193)	1.05 (0.111)	0.0000
IV septal thickness (cm)	1.11 (0.224)	0.97 (0.125)	0.0000
LV mean wall thickness (cm)	1.15 (0.186)	0.99 (0.085)	0.0000
LV mass index (g·m^−2.7^)	52.56 (14.483)	42.02 (8.315)	0.0000
Relative wall thickness	0.5 (0.091)	0.46 (0.071)	0.0264
LV geometry			
Normal geometry (%)	15.9	42.0	
Concentric remodelling (%)	43.9	50.0	0.0000
Eccentric hypertrophy (%)	12.2	8.0	
Concentric hypertrophy (%)	28.0	0	

ARR: aldosterone-to-renin ratio; LV: left ventricular; IV: interventricular.

**Table 3:** Biochemical, hormonal, and echocardiographic characteristics of the subjects according  to aldosterone concentration tertiles.

Variable	Tertile 1	Tertile 2	Tertile 3	*P* value
	(<231 pmol·L^−1^)	(231–276 pmol·L^−1^)	(>276 pmol·L^−1^)	
	(*n* = 45)	(*n* = 44)	(*n* = 44)	
Plasma aldosterone (pmol·L^−1^)	187.6 (28.70)	255.5 (11.41)	315.0 (37.63)	0.0000
Plasma renin (ng·mL^−1^)	19.3 (4.313)	21.6 (5.453)	26.2 (9.674)	0.0032
ARR	10.1 (2.242)	12.5 (3.019)	13.7 (6.228)	0.0002
Age (yrs)	49.3 (10.72)	49.9 (11.53)	48.0 (11.10)	0.7492
Body mass index	26.3 (4.971)	27.4 (4.349)	26.3 (5.136)	0.5103
Plasma sodium (mmol·L^−1^)	138.4 (7.598)	136.3 (6.929)	137.9 (8.173)	0.3514
Plasma potassium (mmol·L^−1^)	4.25 (0.747)	3.87 (0.804)	3.98 (0.798)	0.0974
LV internal diameter (diastole)	4.71 (0.406)	4.76 (0.485)	4.76 (0.489)	0.8697
LV posterior wall thickness (cm)	1.13 (0.159)	1.13 (0.191)	1.14 (0.187)	0.8970
IV septal thickness (cm)	1.05 (0.184)	1.09 (0.219)	1.02 (0.203)	0.2265
Relative wall thickness	0.48 (0.085)	0.48 (0.092)	0.48 (0.082)	0.7365
LV mass index (g·m^−2.7^)	47.1 (9.217)	50.9 (14.59)	47.8 (15.85)	0.3036
Systolic blood pressure (mmHg)	154.1 (21.57)	145.8 (24.15)	152.4 (23.40)	0.1871
Diastolic blood pressure (mmHg)	91.5 (11.86)	85.7 (12.28)	87.5 (10.93)	0.0948
Left atrial diameter (cm)	3.87 (0.421)	3.78 (0.513)	3.89 (0.604)	0.5749
LV geometry				
Normal geometry (%)	22.2	27.9	27.3	
Concentric remodelling (%)	55.6	37.2	45.5	0.5945
Eccentric hypertrophy (%)	11.1	14.0	6.8	
Concentric hypertrophy (%)	11.1	20.9	20.4	

ARR: aldosterone-to-renin ratio; LV: left ventricular; IV: interventricular.

**Table 4:** Correlation coefficients between aldosterone levels, aldosterone-to-renin ratio levels, and  echocardiographic parameters of left ventricular structure.

Variables	Aldosterone	ARR
Plasma aldosterone		
ARR	0.4452**	
Plasma renin	0.3883**	−0.5879**
LV mass index	0.0179	0.0887
Relative wall thickness	0.0043	0.0519
LV mean wall thickness	0.0381	0.1186
LV Internal diameter (diastole)	0.0118	0.0472
LV posterior wall thickness	0.0185	0.1052
IV septal thickness	0.0070	0.0992

ARR: aldosterone-to-renin ratio; LV: left ventricular; IV: interventricular.

**P < 0.0001.

## References

[1] Catanzaro DF, Kurtz TW (2002). Target organ damage in hypertension: mechanisms, prevention, and management. *American Journal of Hypertension*.

[2] Whittle JC, Whelton PK, Seidler AJ, Klag MJ (1991). Does racial variation in risk factors explain black-white differences in the incidence of hypertensive end-stage renal disease?. *Archives of Internal Medicine*.

[3] Tarver-Carr ME, Powe NR, Eberhardt MS (2002). Excess risk of chronic kidney disease among African-American versus white subjects in the United States: a population-based study of potential explanatory factors. *Journal of the American Society of Nephrology*.

[4] Rayner B, Becker P (2006). The prevalence of microalbuminuria and ECG left ventricular hypertrophy in hypertensive patients in private practices in South Africa. *Cardiovascular Journal of South Africa*.

[5] Falkner B (1990). Differences in blacks and whites with essential hypertension: biochemistry and endocrine: state of the art lecture. *Hypertension*.

[6] Alderman MH, Cohen HW, Sealey JE, Laragh JH (2004). Plasma renin activity levels in hypertensive persons: their wide range and lack of suppression in diabetic and in most elderly patients. *American Journal of Hypertension*.

[7] Chrysant SG, Danisa K, Kem DC, Dillard BL, Smith WJ, Frohlich ED (1979). Racial differences in pressure, volume and renin interrelationships in essential hypertension. *Hypertension*.

[8] Osotimehin B, Erasmus RT, Iyun AO, Falase AO, Ahmad Z (1984). Plasma renin activity and plasma aldosterone concentrations in untreated Nigerians with essential hypertension. *African Journal of Medicine and Medical Sciences*.

[9] El-Gharbawy AH, Nadig VS, Kotchen JM (2001). Arterial pressure, left ventricular mass, and aldosterone in essential hypertension. *Hypertension*.

[10] Calhoun DA (2006). Aldosterone and cardiovascular disease: smoke and fire. *Circulation*.

[11] Tomaschitz A, Pilz S (2010). Aldosterone to renin ratio a reliable screening tool for primary aldosteronism?. *Hormone and Metabolic Research*.

[12] Schiller NB, Shah PM, Crawford M (1989). Recommendations for quantitation of the left ventricle by two-dimensional echocardiography. American Society of Echocardiography Committee on Standards, Subcommittee on Quantitation of Two-Dimensional Echocardiograms. *Journal of the American Society of Echocardiography*.

[13] Devereux RB, Alonso DR, Lutas EM (1986). Echocardiographic assessment of left ventricular hypertrophy: comparison to necropsy findings. *American Journal of Cardiology*.

[14] Development Core Team R (2012). *R: A Language and Environment for Statistical Computing*.

[15] Vasan RS, Evans JC, Benjamin EJ (2004). Relations of serum aldosterone to cardiac structure: gender-related differences in the Framingham Heart Study. *Hypertension*.

[16] Stewart AD, Millasseau SC, Dawes M (2006). Aldosterone and left ventricular hypertrophy in Afro-Caribbean subjects with low renin hypertension. *American Journal of Hypertension*.

[17] Edelmann F, Tomaschitz A, Wachter R (2012). Serum aldosterone and its relationship to left ventricular structure and geometry in patients with preserved left ventricular ejection fraction. *European Heart Journal*.

[18] Duprez DA (2004). Is the female heart more sensitive to aldosterone for early remodeling?. *Hypertension*.

[19] Nakahara T, Takata Y, Hirayama Y (2007). Left ventricular hypertrophy and geometry in untreated essential hypertension is associated with blood levels of aldosterone and procollagen type III amino-terminal peptide. *Circulation Journal*.

[20] Schunkert H, Hense H-W, Muscholl M (1997). Associations between circulating components of the renin-angiotensin-aldosterone system and left ventricular mass. *Heart*.

[21] Duprez DA, Bauwens FR, de Buyzere ML (1993). Influence of arterial blood pressure and aldosterone on left ventricular hypertrophy in moderate essential hypertension. *American Journal of Cardiology*.

[22] Malmqvist K, Öhman KP, Lind L, Nyström F, Kahan T (2002). Relationships between left ventricular mass and the renin-angiotensin system, catecholamines, insulin and leptin. *Journal of Internal Medicine*.

[23] Mulè G, Nardi E, Cusimano P (2008). Plasma aldosterone and its relationships with left ventricular mass in essential hypertensive patients with the metabolic syndrome. *American Journal of Hypertension*.

[24] Catena C, Colussi G, Valeri M, Sechi LA (2013). Association of aldosterone with left ventricular mass in hypertension: interaction with plasma fibrinogen levels. *American Journal of Hypertension*.

[25] Weinberg EO, Thienelt CD, Katz SE (1999). Gender differences in molecular remodeling in pressure overload hypertrophy. *Journal of the American College of Cardiology*.

[26] Fox E, Taylor H, Andrew M (2004). Body mass index and blood pressure influences on left ventricular mass and geometry in African Americans: the Atherosclerotic Risk in Communities (ARIC) Study. *Hypertension*.

[27] Guerra F, Mancinelli L, Angelini L (2011). The association of left ventricular hypertrophy with metabolic syndrome is dependent on body mass index in hypertensive overweight or obese patients. *PLoS One*.

[28] Mourad JJ, Hanon O, Girerd X, Boutouyrie P, Safar ME (2000). Effect of hypertension on cardiac mass and radial artery wall thickness. *American Journal of Cardiology*.

[29] Verdecchia P, Reboldi G, Porcellati C (2002). Risk of cardiovascular disease in relation to achieved office and ambulatory blood pressure control in treated hypertensive subjects. *Journal of the American College of Cardiology*.

[30] Missault LH, de Buyzere ML, de Bacquer DD, Duprez DD, Clement DL (2002). Relationship between left ventricular mass and blood pressure in treated hypertension. *Journal of Human Hypertension*.

[31] Verdecchia P, Schillaci G, Guerrieri M (1990). Circadian blood pressure changes and left ventricular hypertrophy in essential hypertension. *Circulation*.

[32] Cuspidi C, Meani S, Valerio C (2007). Age and target organ damage in essential hypertension: role of the metabolic syndrome. *American Journal of Hypertension*.

[33] Negri F, Sala C, Re A, Mancia G, Cuspidi C (2012). Left ventricular geometry and diastolic function in the hypertensive heart: impact of age. *Blood Press*.

[34] Ayodele OE, Alebiosu CO, Akinwusi PO, Akinsola A, Mejiuni A (2007). Target organ damage and associated clinical conditions in newly diagnosed hypertensives attending a tertiary health facility. *Nigerian Journal of Clinical Practice*.

[35] Jin Y, Kuznetsova T, Maillard M (2009). Independent relations of left ventricular structure with the 24-hour urinary excretion of sodium and aldosterone. *Hypertension*.

